# Serum bicarbonate levels and gait abnormalities in older adults: a cross-sectional study

**DOI:** 10.1038/s41598-022-12907-w

**Published:** 2022-06-02

**Authors:** Jim Q. Ho, Joe Verghese, Matthew K. Abramowitz

**Affiliations:** 1 Department of Medicine, Albert Einstein College of Medicine, 1300 Morris Park Avenue, Ullmann 615, Bronx, NY 10461 USA; 2 Department of Neurology, Albert Einstein College of Medicine, Bronx, NY USA; 3 Institute for Aging Research, Albert Einstein College of Medicine, Bronx, NY USA; 4 Diabetes Research Center, Albert Einstein College of Medicine, Bronx, NY USA; 5 Fleischer Institute for Diabetes and Metabolism, Albert Einstein College of Medicine, Bronx, NY USA

**Keywords:** Acid, base, fluid, electrolyte disorders, Chronic kidney disease

## Abstract

Metabolic acidosis is associated with impaired physical function in patients with chronic kidney disease (CKD) and older adults. However, whether acidosis is associated with gait abnormalities has received little attention. In a cohort of 323 community-dwelling adults ≥ 65 years old who underwent quantitative gait analysis, we examined associations of serum bicarbonate with eight individual gait variables. After multivariable adjustment, participants in the lowest bicarbonate tertile (< 25 mEq/L) had 8.6 cm/s slower speed (95% confidence interval [CI] 3.2–13.9), 7.9 cm shorter stride length (95% CI 3.5–12.2), and 0.03 s longer double support time (95% CI 0.002–0.1) compared with those in the middle tertile (25–27 mEq/L). Furthermore, lower bicarbonate levels were associated with more severe gait abnormalities in a graded manner. After further adjustment for possible mediating factors, associations were attenuated but remained significant. Among participants with CKD, associations were of similar or greater magnitude compared with those without CKD. Factor analysis was performed to synthesize the individual gait variables into unifying domains: among the pace, rhythm, and variability domains, lower serum bicarbonate was associated with worse performance in pace. In sum, lower serum bicarbonate was independently associated with worse performance on several quantitative measures of gait among older adults.

## Introduction

Metabolic acidosis is increasingly recognized as a potential cause of physical function impairment^1^. Acidosis is associated with poor muscle strength and with incident functional limitation in older adults^2,3^, and the treatment of moderate-severe acidosis improved lower extremity muscle performance in patients with chronic kidney disease (CKD)^4^. Another aspect of physical function that may be impacted by acidosis is gait. Prior work has identified associations of low serum bicarbonate levels with slow gait speed^2,3^; whether metabolic acidosis also impacts other aspects of gait is unknown. This knowledge gap is important because slow gait is a manifestation of a broader process of gait dysfunction that is associated with elevated fall risk in CKD^5,6^, one that involves spatiotemporal abnormalities such as shortened stride length and gait unsteadiness^5^. If acidosis were associated with the same pattern of abnormalities, this could suggest an important role for acidosis in the development of gait dysfunction in CKD. In addition, low serum bicarbonate could serve as a prognostic marker to detect individuals at risk of declining gait performance.

We hypothesized that metabolic acidosis was associated with spatiotemporal gait abnormalities and tested this hypothesis in a cohort of community-dwelling older adults using serum bicarbonate as a marker of acid–base homeostasis. We also hypothesized that associations of serum bicarbonate with gait abnormalities would be strongest among individuals in whom low bicarbonate most likely represented metabolic acidosis. Therefore, given that pH measurements were not available, to test whether our results were truly reflective of metabolic acidosis, we also performed analyses stratified by CKD status, because among individuals with CKD, low serum bicarbonate is more likely to represent metabolic acidosis^7^.

## Results

### Participant characteristics

**Figure 1:**
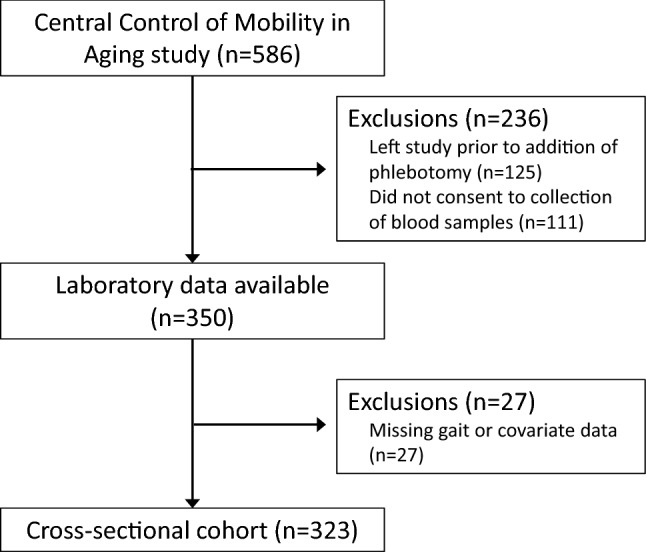
Flow diagram of included study participants.

Three hundred twenty-three participants were included in the analysis (Fig. 1), 131 (41%) of whom had CKD. There were 112 participants in the low bicarbonate tertile (< 25 mEq/L, median 23 [interquartile range (IQR) 22–24]), 144 participants in the middle bicarbonate tertile (25–27 mEq/L, median 26 [IQR 25–27]), and 67 participants in the high bicarbonate tertile (> 27 mEq/L, median 28 [IQR 28–29]) (Table 1; sample size differs across tertiles because serum bicarbonate is only measured in integer values). Forty-six (14%) participants had serum bicarbonate < 23 mEq/L. Participants with lower bicarbonate had higher prevalence of neuropathy, greater medication use, lower estimated glomerular filtration rate (eGFR), and higher prevalence of CKD. Compared with participants in the low and high bicarbonate tertiles, those in the middle tertile had faster gait speed, longer stride length, and shorter time in the stance and double-support phase.

**Table 1:** Participant characteristics by serum bicarbonate tertile.

Characteristic	Bicarbonate tertile (mEq/L)			
	< 25 (n = 112)	25–27 (n = 144)	> 27 (n = 67)	*P* value
Age (year)	78 ± 7	77 ± 6	78 ± 7	0.29
Women, n (%)	60 (54)	73 (51)	40 (60)	0.47
White, n (%)	88 (79)	110 (76)	54 (81)	0.78
**Education, n (%)**				0.70
High school or less	37 (33)	41 (28)	24 (36)	
College	51 (46)	64 (44)	26 (39)	
Postgraduate	24 (21)	39 (27)	17 (25)	
Body mass index, kg/m^2^	28 (25–33)	27 (24–32)	27 (24–30)	0.08
**Comorbidities (global health score), n (%)**				0.10
0	16 (14)	25 (17)	11 (16)	
1	23 (21)	49 (34)	25 (37)	
2	44 (39)	47 (42)	19 (28)	
3 +	29 (26)	23 (16)	12 (18)	
Hypertension, n (%)	76 (68)	85 (59)	35 (52)	0.10
Diabetes, n (%)	28 (25)	22 (15)	13 (19)	0.15
Neuropathy, n (%)	20 (18)	10 (7)	4 (6)	0.01
Cardiovascular disease, n (%)	18 (16)	17 (12)	9 (13)	0.61
Emphysema/COPD, n (%)	12 (11)	7 (5)	3 (4)	0.13
**Smoking cigarettes, n (%)**				0.32
Never	53 (47)	60 (42)	37 (55)	
Former	53 (47)	78 (54)	29 (43)	
Current	6 (5)	6 (4)	1 (1)	
Medication count	2.0 ± 0.9	1.8 ± 0.9	1.7 ± 1.0	0.03
Diuretic use, n (%)	15 (13)	24 (17)	12 (18)	0.67
BUN (mg/dL)	21 (16.5–27)	19 (16–24)	18 (16–22)	0.06
eGFR (mL/min per 1.73 m^2^)	57 (44–73)	65 (54–75)	69 (57–79)	0.002
CKD, n (%)	58 (52)	50 (35)	23 (34)	0.01
Bicarbonate (mEq/L)	23 (22–24)	26 (25–27)	28 (28–29)	< 0.001
**Gait variables**				
Speed (cm/s)	89 ± 22	101 ± 22	98 ± 26	< 0.001
Stride length (cm)	107 ± 21	118 ± 19	115 ± 21	< 0.001
Double support time (s)	0.41 ± 0.11	0.37 ± 0.09	0.39 ± 0.11	0.004
Stance time (s)	0.82 ± 0.13	0.78 ± 0.11	0.79 ± 0.14	0.048
Swing time (s)	0.40 ± 0.05	0.40 ± 0.04	0.40 ± 0.05	0.50
Cadence (steps/min)	100 ± 12	103 ± 12	102 ± 13	0.24
Stride length SD (cm)	3 (2–4)	3 (2–5)	4 (2–5)	0.68
Swing time SD (s)	0.02 ± 0.01	0.02 ± 0.01	0.02 ± 0.01	0.26

Data are expressed as mean ± SD, median (interquartile range), or count (percentage). Baseline comparisons among bicarbonate tertiles were conducted using one-way analysis of variance (ANOVA) or Kruskal–Wallis test as appropriate for continuous variables and Pearson’s chi-squared test for categorical variables. Homoscedasticity was assessed using Bartlett’s test.

CKD was defined as eGFR < 60 mL/min per 1.73 m^2^. N = 323 total.

*BUN* blood urea nitrogen, *CKD* chronic kidney disease, *COPD* chronic obstructive pulmonary disease, *eGFR* estimated glomerular filtration rate, *SD* standard deviation.

### Association of serum bicarbonate with quantitative gait markers

Compared with participants in the bicarbonate 25–27 mEq/L (middle) tertile, those in the < 25 mEq/L (low) tertile had 8.6 cm/s (95% confidence interval [CI] 3.2 to 13.9) slower gait speed, 7.9 cm (95% CI 3.5–12.2) shorter stride length, and 0.03 s (95% CI 0.002–0.1) longer double support time after multivariable adjustment (Table 2). There were no significant associations with the other gait variables. The > 27 mEq/L (high) tertile was not significantly associated with differences in any of the gait variables.

**Table 2:** Associations of serum bicarbonate tertiles with gait variables.

Gait variable	Bicarbonate tertile (mEq/L)				
	< 25 (n = 112)		25–27 (n = 144)	> 27 (n = 67)	
	Coefficient (95% CI)	*P* value		Coefficient (95% CI)	*P* value
Speed (cm/s)	− 8.6 (− 13.9 to − 3.2)	0.002	Ref	− 2.4 (− 8.5 to 3.7)	0.44
Stride length (cm)	− 7.9 (− 12.2 to − 3.5)	< 0.001	Ref	− 1.9 (− 6.8 to 3.1)	0.46
Double support time (s)	0.03 (0.002–0.1)	0.04	Ref	0.02 (− 0.005 to 0.1)	0.11
Stance time (s)	0.03 (− 0.005 to 0.1)	0.10	Ref	0.03 (− 0.01 to 0.1)	0.15
Swing time (s)	− 0.001 (− 0.01 to 0.01)	0.82	Ref	− 0.002 (− 0.01 to 0.01)	0.79
Cadence (steps/min)	− 2.1 (− 5.1 to 1.0)	0.18	Ref	− 1.6 (− 5.1 to 1.8)	0.36
Stride length SD (cm)	− 0.1 (− 0.6 to 0.4)	0.67	Ref	0.4 (− 0.2 to 0.9)	0.19
Swing time SD (s)	0.0003 (− 0.003 to 0.003)	0.87	Ref	0.002 (− 0.002 to 0.01)	0.27

Multivariable linear regression adjusting for age, sex, race, education, smoking status, body mass index (BMI), number of comorbidities, number of medications, diuretic use, diagnosis of neuropathy, cardiovascular disease, hypertension, diabetes, emphysema or chronic obstructive pulmonary disease (COPD), blood urea nitrogen (BUN), and estimated glomerular filtration rate (eGFR) was performed. The reference group was serum bicarbonate 25–27 mEq/L (middle tertile).

*Ref* reference group, *SD* standard deviation.

N = 323 total.

To synthesize individual gait characteristics into unifying domains, factor analysis was performed on the eight gait variables. Factor analysis examines interrelationships between correlated variables to identify underlying constructs, in this case unifying characteristics of the gait cycle (gait domains). This produced 3 gait domains explaining 89% of the variance (Table 3). The pace domain (factor 1) accounted for 46% of the variance and loaded mainly on gait speed, stride length, and time in the stance and double-support phases. The rhythm domain (factor 2) accounted for 27% of the variance and loaded primarily in time in the swing phase and cadence. The variability domain (factor 3) accounted for 17% of the variance and loaded heavily in stride length standard deviation (SD) and swing time SD. We then examined associations of serum bicarbonate with each of these gait domains: compared with those in the middle bicarbonate tertile, participants in the < 25 mEq/L tertile had 0.3 SD (95% CI 0.1–0.6) worse performance in the pace domain but not in the other two domains; the gait variables on which the pace domain loaded most heavily were consistent with the primary findings using the 8 individual gait variables.

**Table 3:** Associations of serum bicarbonate tertiles with gait domains using factor analysis.

Gait variable	Gait domain				
	Pace (SD)	Rhythm (SD)	Variability (SD)		
	**Rotated factor loadings**				
Speed (cm/s)	**0.95**	− 0.18	− 0.14		
Stride length (cm)	**0.91**	0.28	− 0.13		
Double support time (s)	**− 0.89**	0.32	0.10		
Stance time (s)	**− 0.77**	0.59	0.10		
Swing time (s)	0.07	**0.95**	0.05		
Cadence (steps/min)	0.58	**− 0.79**	− 0.11		
Stride length SD (cm)	− 0.01	0.02	**0.94**		
Swing time SD (s)	− 0.49	0.15	**0.61**		
Variance explained (%)	46	27	17		

Gait domain	Bicarbonate tertile (mEq/L)				
	< 25 (n = 112)		25–27 (n = 144)	> 27 (n = 67)	
	Coefficient (95% CI)	*P* value		Coefficient (95% CI)	*P* value
Pace (SD)	− 0.3 (− 0.6 to − 0.1)	0.003	Ref	− 0.1 (− 0.4 to 0.1)	0.32
Rhythm (SD)	− 0.1 (− 0.3 to 0.2)	0.60	Ref	0.02 (− 0.3 to 0.3)	0.89
Variability (SD)	− 0.1 (− 0.3 to 0.2)	0.59	Ref	0.2 (− 0.1 to 0.5)	0.15

Top: Rotated factor loadings of eight gait variables using factor analysis. Factor analysis was performed using the principal component method with varimax rotation. The highest loading variables are in bold. Bottom: Multivariable linear regression adjusting for age, sex, race, education, smoking status, body mass index (BMI), number of comorbidities, number of medications, diuretic use, diagnosis of neuropathy, cardiovascular disease, hypertension, diabetes, emphysema or chronic obstructive pulmonary disease (COPD), blood urea nitrogen (BUN), and estimated glomerular filtration rate (eGFR) was performed. The reference group was serum bicarbonate 25–27 mEq/L (middle tertile).

N = 323 total.

*CI* confidence interval, *Ref* reference group, *SD* standard deviation.

**Figure 2:**
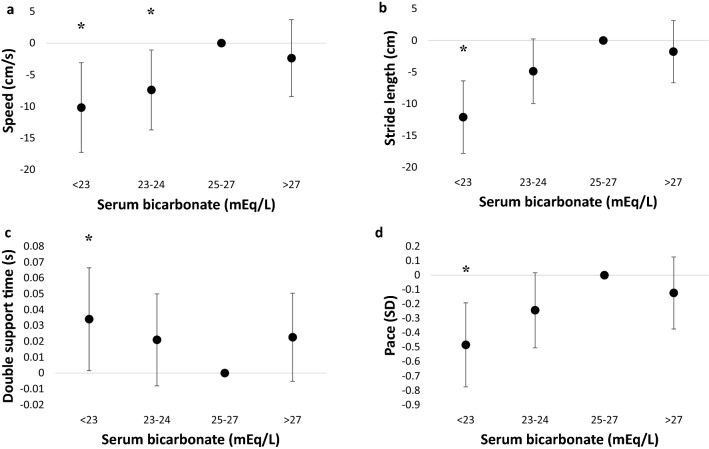
Lower serum bicarbonate is associated with progressively greater gait disturbances. Multivariable linear regression was performed on (**a**) gait speed, (**b**) stride length, (**c**) double support time, and (**d**) pace. The reference group was serum bicarbonate 25–27 mEq/L (middle tertile). Models were adjusted for age, sex, race, education, smoking status, body mass index (BMI), number of comorbidities, number of medications, diuretic use, diagnosis of neuropathy, cardiovascular disease, hypertension, diabetes, emphysema or chronic obstructive pulmonary disease (COPD), blood urea nitrogen (BUN), and estimated glomerular filtration rate (eGFR). Error bars denote 95% confidence intervals. Asterisks denote *P* < 0.05. Serum bicarbonate < 23 mEq/L (n = 46), 23–24 mEq/L (n = 66), 25–27 mEq/L (n = 144), > 27 mEq/L (n = 67). *SD* standard deviation. N = 323 total.

To determine whether there was a graded association of lower serum bicarbonate levels with severity of gait deficits, the bicarbonate < 25 mEq/L tertile was further divided into subgroups of bicarbonate 23–24 mEq/L and < 23 mEq/L. Compared with the bicarbonate 25–27 mEq/L tertile, participants in the 23–24 mEq/L and < 23 mEq/L subgroups had 7.4 cm/s (95% CI 1.1–13.7) and 10.2 cm/s (95% CI 3.1 to 17.3) slower gait speed, respectively (Fig. 2). Participants in the bicarbonate < 23 mEq/L subgroup also had 12.1 cm (95% CI 6.4–17.8) shorter stride length, 0.03 s (95% CI 0.002–0.07) longer double support time, and 0.5 SD (95% CI 0.2–0.8) worse performance in the pace domain than those in the 25–27 mEq/L tertile.

We further examined the association between continuous bicarbonate levels and gait markers using linear splines (Table 4). Every 1 mEq/L serum bicarbonate below 26 mEq/L was associated with 1.9 cm/s (95% CI 0.5–3.3) slower gait speed, 2.1 cm (95% CI 1.0–3.3) shorter stride length, 0.007 s (95% CI 0.0002–0.013) longer double support time; and, consistent with these findings, 0.09 SD (95% CI 0.03–0.15) worse performance in the pace domain. When bicarbonate level was above 26 mEq/L, bicarbonate was associated only with longer double support time (0.011 s [95% CI 0.0005–0.021] per 1 mEq/L higher serum bicarbonate).

**Table 4:** Associations of continuous serum bicarbonate levels with gait markers using linear splines.

Gait marker	Bicarbonate level			
	Per 1 mEq/L lower than 26 mEq/L		Per 1 mEq/L higher than 26 mEq/L	
	Coefficient (95% CI)	*P* value	Coefficient (95% CI)	*P* value
	**All participants (n = 323)**			
Speed (cm/s)	− 1.9 (− 3.3 to − 0.5)	0.01	− 1.2 (− 3.5 to 1.0)	0.28
Stride length (cm)	− 2.1 (− 3.3 to − 1.0)	< 0.001	− 0.9 (− 2.8 to 0.9)	0.32
Double support time (s)	0.007 (0.0002 to 0.013)	0.04	0.011 (0.0005 to 0.021)	0.04
Pace (SD)	− 0.09 (− 0.15 to − 0.03)	0.004	− 0.06 (− 0.16 to 0.03)	0.18
	**CKD (n = 131)**			
Speed (cm/s)	− 2.8 (− 5.1 to − 1.0)	0.02	− 0.9 (− 5.3 to 3.4)	0.67
Stride length (cm)	− 3.1 (− 5.0 to − 1.1)	0.002	− 0.8 (− 4.5 to 2.9)	0.67
Double support time (s)	0.009 (− 0.002 to 0.019)	0.12	0.011 (− 0.010 to 0.032)	0.31
Pace (SD)	− 0.12 (− 0.22 to − 0.02)	0.02	− 0.07 (− 0.26 to 0.13)	0.50
	**Non-CKD (n = 192)**			
Speed (cm/s)	− 1.1 (− 3.2 to 1.0)	0.29	− 1.1 (− 3.9 to 1.7)	0.42
Stride length (cm)	− 1.0 (− 2.6 to 1.0)	0.22	− 1.0 (− 2.8 to 1.5)	0.56
Double support time (s)	0.006 (− 0.003 to 0.015)	0.22	0.011 (− 0.002 to 0.023)	0.10
Pace (SD)	− 0.06 (− 0.14 to 0.02)	0.14	− 0.06 (− 0.17 to 0.05)	0.30

*CI* confidence interval, *CKD* chronic kidney disease, *SD* standard deviation.

Multivariable linear spline regression adjusting for age, sex, race, education, smoking status, body mass index (BMI), number of comorbidities, number of medications, diuretic use, diagnosis of neuropathy, cardiovascular disease, hypertension, diabetes, emphysema or chronic obstructive pulmonary disease (COPD), blood urea nitrogen (BUN), and estimated glomerular filtration rate (eGFR) was performed. The knot was placed at serum bicarbonate 26 mEq/L. CKD was defined as eGFR < 60 mL/min per 1.73 m^2^. *P* values for interaction between continuous serum bicarbonate below 26 mEq/L and eGFR: gait speed (*P* = 0.05), stride length (*P* = 0.01), double support time (*P* = 0.04), pace (*P* = 0.02). *P* values for interaction between continuous serum bicarbonate above 26 mEq/L and eGFR: gait speed (*P* = 0.52), stride length (*P* = 0.59), double support time (*P* = 0.28), pace (*P* = 0.52).

Next, we investigated whether other aspects of physical function could explain the associations between serum bicarbonate levels and these gait markers. After adjusting for muscle strength, cognitive function, somatosensory sensitivity, and balance, the effect estimates were attenuated but remained significant (Supplementary Table S1; percentage attenuated for bicarbonate < 25 mEq/L tertile: gait speed 20%, stride length 20%, double support time 17%, pace 17%).

### Serum bicarbonate, kidney function, and gait

**Figure 3:**
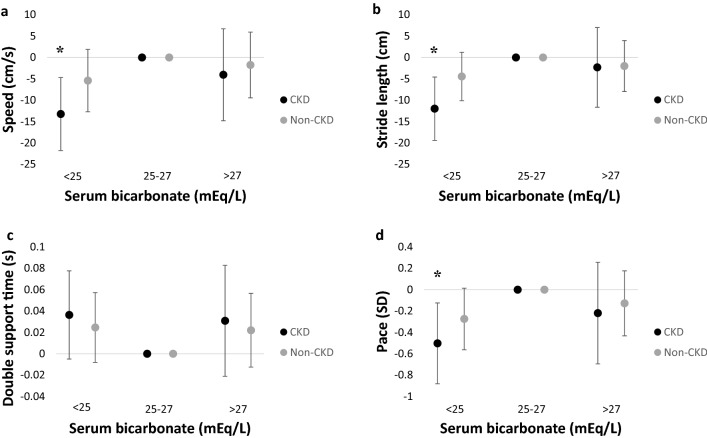
Associations of serum bicarbonate tertiles with gait markers by chronic kidney disease (CKD) status. Multivariable linear regression was performed on (**a**) gait speed, (**b**) stride length, (**c**) double support time, and (**d**) pace for CKD (n = 131) and non-CKD (n = 192) participants. The reference group was serum bicarbonate 25–27 mEq/L (middle tertile). CKD was defined as estimated glomerular filtration rate (eGFR) < 60 mL/min per 1.73 m^2^. Models were adjusted for age, sex, race, education, smoking status, body mass index (BMI), number of comorbidities, number of medications, diuretic use, diagnosis of neuropathy, cardiovascular disease, hypertension, diabetes, emphysema or chronic obstructive pulmonary disease (COPD), blood urea nitrogen (BUN), and eGFR. Error bars denote 95% confidence intervals. Asterisks denote *P* < 0.05. Serum bicarbonate < 25 mEq/L (CKD n = 58, non-CKD n = 54), 25–27 mEq/L (CKD n = 50, non-CKD = 94), > 27 mEq/L (CKD n = 23, non-CKD n = 44). *CKD* chronic kidney disease, *SD* standard deviation. N = 323 total.

We proceeded to investigate whether the observed associations were modified by CKD status. There were 131 participants with CKD and 192 participants without CKD. Analyses stratified by CKD status suggested associations of greater magnitude among participants with CKD compared with those without (Fig. 3). These analyses were confirmed by linear spline analysis (Table 4). No effect modification was observed by CKD status when bicarbonate levels were above 26 mEq/L. Among participants without CKD, associations were of smaller magnitude and not statistically significant. The *P* values for interaction between continuous bicarbonate below 26 mEq/L and eGFR were: gait speed (*P* = 0.05), stride length (*P* = 0.01), double support time (*P* = 0.04), and the pace domain (*P* = 0.02).

## Discussion

In a cohort of 323 community-dwelling older adults, lower serum bicarbonate levels were independently associated with several aspects of gait dysfunction, including slower gait speed, shorter stride length, and longer time in the double support phase of the gait cycle. In combination, these translated into worse performance in the pace domain of gait. Furthermore, there were graded associations between lower bicarbonate levels and more severe gait abnormalities. Our analyses also revealed that CKD modified the association between lower bicarbonate levels and gait abnormalities; effect estimates were of greater magnitude among those with CKD. These results provide the first evidence that metabolic acidosis may be associated with a broad pattern of gait dysfunction in older adults, especially those with CKD.

These findings could have clinical implications for the growing number of older adults with CKD^8^. In this population, gait dysfunction is an important cause of falls, ultimately leading to disability and loss of independence^5,9,10^. Specifically, slow gait speed, short stride length, and worse performance on the pace domain are associated with functional limitation, falls, cognitive dysfunction, and mortality^11–13^. Importantly, metabolic acidosis is a common yet treatable complication of CKD^14^. The treatment of acidosis has improved physical function in some studies but not others; however, no prior study assessed gait quality as an outcome^4,15–17^.

Consistent with our findings, prior work found associations of low serum bicarbonate with slow gait speed^2,3^. Our study extends this body of evidence by demonstrating that low bicarbonate levels are associated with a pattern of gait dysfunction that is remarkably similar to the pattern we previously described in association with CKD^5^. Thus, our results indicate that metabolic acidosis may be associated with gait dysfunction in older adults with CKD; whether this represents a causal relationship remains to be determined. Alternatively, low serum bicarbonate may identify a subset of CKD patients at high risk for gait abnormalities, or acidosis and gait dysfunction may jointly identify a subset of older adults at risk for poor outcomes. Future prospective studies will be needed to test these hypotheses.

The mechanism linking low bicarbonate levels with gait dysfunction requires additional study. Associations were similar among the subgroup with CKD as in the full cohort but appeared to be of greater magnitude in those with CKD. As low serum bicarbonate is more likely to represent metabolic acidosis in the setting of reduced eGFR, this suggests that the associations we observed are less likely confounded by respiratory alkalosis and truly represent a link between metabolic acidosis and gait abnormalities^7,18,19^. Among the subgroup without CKD, it is likely that in some individuals low serum bicarbonate was due to respiratory alkalosis, which may not be associated with gait dysfunction; this could explain the stronger associations observed in the subgroup with CKD. Another likely explanation is that the distribution of serum bicarbonate was lower among participants with CKD compared to those without; therefore, greater severity of acidosis could have resulted in stronger associations.

To better understand our findings, we considered several pathways through which acidosis could impact gait. Adverse effects of metabolic acidosis on skeletal muscle are well-described, and associations with cognitive dysfunction were recently reported; both skeletal muscle and cognitive effects could contribute to gait dysfunction^1,20^. Similarly, alterations of acid–base balance could impact neuronal signaling, thereby affecting balance and peripheral nerve function^21,22^. However, associations of serum bicarbonate with gait parameters remained significant after adjustment for measures of each pathway, suggesting that an additional explanation, or more precise measures of these putative mechanisms, is required.

This study had several limitations. A single serum bicarbonate level was measured to determine acid–base status^2^. Arterial blood samples were not collected, so a complete acid–base status determination was not possible. A single serum creatinine measurement was used to define CKD, which has been done previously^23^. No data were collected for albuminuria and cystatin C. Since serum creatinine is correlated with muscle mass, a cystatin C-based eGFR would have been informative. Since elevated dietary acid is associated with lower serum bicarbonate levels, dietary data would have been informative as well^24^. However, adjustments for blood urea nitrogen as a marker of dietary protein intake did not change our results. Given that comorbidities were self-reported, residual confounding is a possibility. The cross-sectional design means that reverse causality cannot be excluded. Finally, several aspects of sampling could have resulted in a non-representative cohort: laboratory results were not available in all participants, and the low prevalence of comorbidities and low number of medications suggest that a relatively healthy group of participants was recruited, which may reduce the study’s generalizability.

In sum, among community-dwelling older adults, metabolic acidosis (as determined by lower serum bicarbonate) was associated with gait dysfunction. The severity of gait dysfunction was directly associated with the severity of acidosis, and the pattern of gait abnormalities was similar to that recently described in association with CKD. Future studies should explore mechanisms through which acidosis may affect gait and the impact of correcting acidosis on gait parameters.

## Methods

### Study population

Mobility and disability in older adults were investigated by the Central Control of Mobility in Aging (CCMA) study^25^. Between June 2011 and October 2017, recruitment of residents who were age ≥ 65 years occurred from population lists of lower Westchester County, New York. Potential participants were excluded if they could not speak English, had dementia, had severe vision or hearing impairment that would prevent completion of the clinical assessments, had active or history of severe neurologic or psychiatric conditions, had recent or anticipated medical procedures impacting mobility, or were on hemodialysis. In-person evaluations occurred every year. The CCMA study protocols were approved by the Institutional Review Board of the Albert Einstein College of Medicine (IRB # 2010-224). The study was performed in accordance with the Declaration of Helsinki. All participants provided written informed consent prior to enrolment.

A subsample of participants with blood samples collected from the CCMA study is used in the present analyses (Fig. 1). The sample selection was explained previously^5^. Between July 2013 (when the CCMA study began collecting blood samples) and June 2017, this subsample of participants had study visits with laboratory data collected. Laboratory data, including serum bicarbonate and creatinine, were measured in the clinical laboratory of Montefiore Medical Center. In the same visit, gait was analyzed, and information was gathered about comorbidities and medications. Participants were excluded if the gait analysis and laboratory collection were not at the same visit or if they had missing data for covariates of interest. Participants with laboratory data had no significant differences in age, sex, race, comorbidity burden, or gait speed compared with those without laboratory data^5^.

### Study design

This was an observational cross-sectional study. Participant data such as demographics, comorbidities, and medications were acquired via standardized medical questionnaires. Cardiovascular disease was defined as a history of congestive heart failure, stent or angioplasty, myocardial infarction, coronary artery bypass graft, or angina diagnosed by a physician. Neurologic examinations were conducted by study clinicians to determine the presence or absence of peripheral neuropathy (stocking distribution sensory loss, absent ankle reflexes or foot drop).

### Assessments

The protocols used by research assistants for the Michigan Neuropathy Score, quadriceps strength, grip strength, repeated chair stands, Repeatable Battery for the Assessment of Neuropsychological Status (RBANS), vibration threshold, and unipedal stance were previously described^5^. The RBANS was used to assess general mental status^26^.

#### Neuropathy

Neuropathic symptoms (range 0–15, lower better) were evaluated with the Michigan Neuropathy Screening Instrument, which is a validated measure of neuropathy severity^27,28^. A Vibratron II (Physitemp Instruments, Clifton, NJ) was used to measure vibration threshold (somatosensory sensitivity) in microns^29^. A standardized forced-choice protocol was used, and has been described before^30^.

#### Physical function testing

The Short Physical Performance Battery, which includes gait speed, chair rise, and balance tasks (range 0–12, higher better), tested lower extremity function^31^. The time participants could stand on one self-selected leg without support for a maximum of 30 s (unipedal stance test) was measured as a sensitive test of balance^32^. A Jamar Dynamometer was used to assess grip strength, which was the maximum voluntary contraction in the dominant hand, with the highest value of three trials recorded. A dynamometer (Lafayette Manual Muscle Test System; Lafayette Instrument Company, Lafayette, IN) was used to test knee extensor strength with 90 degrees of knee flexion, with the highest value of three trials recorded.

#### Laboratory testing

Serum bicarbonate was measured enzymatically using an assay that measures total carbon dioxide content (normal range 22–29 mEq/L). The creatinine-based Chronic Kidney Disease Epidemiology Collaboration equation was used to calculate the eGFR^33^. A modified kinetic Jaffe reaction was used to quantify the serum creatinine. CKD was defined as an eGFR < 60 mL/min per 1.73 m^2^ as described in this cohort previously^5,34^.

### Quantitative gait analysis

The gait analysis has been described previously^5^. Quantitative gait cycle characteristics were assessed using a 20-foot computerized walkway (GAITRite; CIR Systems, Franklin, NJ). Participants were asked to ambulate at their usual pace on the walkway while wearing comfortable footwear and without any attached devices or sensors. Start and stop points were marked on the floor; these included 3 feet from the walkway edge for acceleration and deceleration. Gait speed, stride length, cadence, and time in the double support, swing, and stance phases of the gait cycle were analyzed, as well as the stride length and swing time standard deviations as measures of variability. The mean of each parameter from two walking trials was computed. The GAITRite system has been validated and determined to be reliable for quantitative gait analysis^35,36^. In the elderly population, this method has been shown to be highly reliable and valid for numerous outcomes^13,37^.

### Statistical analyses

The participants were categorized into tertiles (three quantiles) of serum bicarbonate. The low and high tertiles were compared with the middle tertile (reference group), because a nonlinear association between bicarbonate and gait was found in the past^2^. Baseline comparisons among bicarbonate tertiles were conducted using one-way analysis of variance (ANOVA) or Kruskal–Wallis test as appropriate for continuous variables and Pearson’s chi-squared test for categorical variables. Homoscedasticity was assessed using Bartlett’s test. Linear regression models were used to examine associations between bicarbonate and quantitative gait markers; unstandardized coefficients and 95% CIs were computed. Covariates determined a priori based on prior studies: age, sex, race, education, smoking status, body mass index (BMI), number of comorbidities, number of medications, diuretic use, diagnosis of neuropathy, cardiovascular disease, hypertension, diabetes, emphysema or chronic obstructive pulmonary disease (COPD), blood urea nitrogen (BUN), and eGFR. Based on our prior work, eGFR was modelled by creating linear splines with a knot at eGFR = 60 mL/min per 1.73 m^2^. Based on the graphical appearance of the relationship between bicarbonate levels and gait markers, linear splines with a knot at bicarbonate = 26 mEq/L (median of the middle tertile) were created^5^. As a secondary analysis, factor analysis was performed to consolidate the individual gait variables into statistically independent gait domains, using the principal component method with varimax rotation as previously done in an elderly, community-dwelling cohort^13^. A maximum of 3 factors to be retained was specified. Factor loadings with the individual gait variables were examined to identify the gait construct represented. Each factor was then included as the dependent variable in the regression models previously described. Effect modification between bicarbonate levels and eGFR was evaluated with multiplicative interaction terms. *P* < 0.05 was considered statistically significant. Stata/MP version 13.0 (StataCorp, College Station, TX) was used for all analyses.

## Supplementary Information

Supplementary Table S1.

## Data Availability

The datasets analyzed during the current study are available from the corresponding author on reasonable request.
