# Supplementary material for: Serum bicarbonate levels and gait abnormalities in older adults: a cross-sectional study
Source: Sci Rep. 2022 Jun 2;12:9171. doi: 10.1038/s41598-022-12907-w (PMC9163170; doi:10.1038/s41598-022-12907-w)
Supplement: Supplementary file 1 — Supplementary Table S1. [file 41598_2022_12907_MOESM1_ESM.pdf]

SUPPLEMENTARY INFORMATION

Supplementary Table S1. Associations of serum bicarbonate tertiles with gait markers after including possible mediators in multivariable model.

Supplementary Table S1. Associations of serum bicarbonate tertiles with gait markers after including possible mediators in multivariable model.

Gait marker	Bicarbonate tertile (mEq/L)				
	<25 (n=62)		25–27 (n=96)	>27 (n=38)	
	Coefficient (95% CI)	*P* value		Coefficient (95% CI)	*P* value
	Without mediators				
Speed (cm/s)	-8.7 (-15.5 to -2.0)	0.01	Ref	-3.0 (-10.5 to 4.5)	0.43
Stride length (cm)	-7.4 (-13.3 to -1.6)	0.01	Ref	-2.5 (-9.0 to 4.1)	0.46
Double support time (s)	0.04 (0.01 to 0.07)	0.003	Ref	0.03 (-0.0003 to 0.06)	0.052
Pace (SD)	-0.4 (-0.7 to -0.1)	0.003	Ref	-0.2 (-0.5 to 0.1)	0.17
	With mediators				
Speed (cm/s)	-7.0 (-13.2 to -0.8)	0.03	Ref	-3.5 (-10.4 to 3.3)	0.31
Stride length (cm)	-6.0 (-11.2 to -0.7)	0.03	Ref	-2.8 (-8.7 to 3.0)	0.34
Double support time (s)	0.03 (0.01 to 0.1)	0.01	Ref	0.03 (0.01 to 0.1)	0.02
Pace (SD)	-0.3 (-0.6 to -0.1)	0.01	Ref	-0.2 (-0.5 to 0.03)	0.09

Multivariable linear regression adjusting for age, sex, race, education, smoking status, body mass index (BMI), number of comorbidities, number of medications, diuretic use, diagnosis of neuropathy, cardiovascular disease, hypertension, diabetes, emphysema or chronic obstructive pulmonary disease (COPD), blood urea nitrogen (BUN), estimated glomerular filtration rate (eGFR), and with or without mediators (Michigan Neuropathy Score, quadriceps strength, grip strength, repeated chair stands, Repeatable Battery for the Assessment of Neuropsychological Status [RBANS], vibration threshold, unipedal stance) was performed. The reference group was serum bicarbonate 25–27 mEq/L (middle tertile). CI, confidence interval. Ref, reference group. SD, standard deviation. N=196 total (participants with mediator data).
